# The Role of Vitamins in Mitigating the Effects of Various Stress Factors in Pigs Breeding

**DOI:** 10.3390/ani14081218

**Published:** 2024-04-18

**Authors:** Grzegorz Smołucha, Anna Steg, Maria Oczkowicz

**Affiliations:** Department of Animal Molecular Biology, National Research Institute of Animal Production, ul. Krakowska 1, 32-083 Balice, Poland; anna.steg@iz.edu.pl (A.S.); maria.oczkowicz@iz.edu.pl (M.O.)

**Keywords:** vitamin, nutrition, antioxidants, stress, pig

## Abstract

**Simple Summary:**

Pigs are exposed to various stressors, which can disrupt their internal balance and compromise their well-being. Stress can adversely affect animal performance, fertility, and immunity, leading to economic losses for farmers. Incorporating antioxidants in pig breeding can counteract those changes. This review presents studies investigating the impact of additional vitamin supplementation on stress reduction in pigs. It highlights the potential benefits of these dietary antioxidants in maintaining pig health.

**Abstract:**

Good practices in farm animal care are crucial for upholding animal well-being, efficiency, and health. Pigs, like other farm animals, are exposed to various stressors, including environmental, nutritional, chemical, psychological, physiological, and metabolic stressors, which can disrupt their internal balance and compromise their well-being. Oxidative stress can adversely affect animal performance, fertility, and immunity, leading to economic losses for farmers. Dietary considerations are hugely important in attaining these objectives. This paper reviews studies investigating the impact of additional vitamin supplementation on stress reduction in pigs. Vitamin A can be beneficial in counteracting viral and parasitic threats. Vitamin B can be a potential solution for reproductive issues, but it might also be beneficial in reducing the effects of inappropriate nutrition. Vitamin C plays a vital role in reducing the effects of heat stress or exposure to toxins in pigs. Vitamin D proves to be beneficial in addressing stress induced mostly by infections and weaning, while vitamin E has been shown to mitigate the effects of toxins, heat stress, or transport stress. This review highlights the potential benefits of these dietary antioxidants in maintaining pig health, enhancing productivity, and counteracting the adverse effects of various stressors. Understanding the role of vitamins in pig nutrition and stress management is vital for optimising farm animal welfare and production efficiency.

## 1. Introduction

Farm animals’ welfare, productivity, and health are intricately tied to farm animal husbandry practices [1]. Keeping animals in optimal condition is crucial for efficiently delivering quality animal products and safe food [2]. Nutrition has a significant impact on health, animal welfare, and production performance. In recent years, much attention has been paid to the problem of animals being exposed to oxidative stress. It is emphasised that feed rations should contain adequate amounts of antioxidants that reduce damage to cellular macromolecules [3].

Like other animals, pigs are exposed to various types of stressors: environmental (extreme temperatures [4] and transportation [5]), nutritional (nutritional adjustments), chemical (pesticides, disinfectants, medications, toxins, or pathogens), psychological (integration into new social groups, fear or restraint, or social separation during weaning), physiological (diseases [6] and stressors related to pregnancy or lactation), and metabolic (ketosis, acidosis, and hypocalcemia), through high performance in intensive livestock farms (management practices collectively contributing to heightened stress levels) [7,8] (graphical abstract in Supplementary Materials Table S1). Prolonged exposure to a variety of stress-inducing conditions disrupts homeostasis, prompting the development of oxidative stress perceived as detrimental to the animal’s well-being [9,10,11].

Oxidative stress is defined as an imbalance between producing Reactive Oxygen Species (ROS)/Reactive Nitrogen Species (RNS) and the body’s ability to detoxify or repair the damage [12]. ROS are highly reactive molecules containing oxygen, such as free radicals and peroxides naturally produced in the body as byproducts of normal metabolism [13]. They play essential roles in cell signalling and homeostasis. However, when ROS levels become excessive due to stress factors and an imbalance arises between the production of ROS and the antioxidant defence system, they can overwhelm the body’s antioxidant defences and cause damage to proteins, lipids, and DNA. This oxidative damage can lead to cellular dysfunction, inflammation, and tissue injury, contributing to various health problems and diseases [14,15,16,17]. RNS species are a group of molecules formed during the reaction between nitric oxide (NO) and other reactive oxygen species (ROS) in the body [18]. RNS play a significant role in various physiological and pathophysiological processes. They can cause posttranslational modifications of proteins, such as nitration of tyrosine residues, which can impact protein function [19]. RNS-induced nitration is often referred to as nitrosative stress [20]. Overall, RNS have diverse effects on different body parts and can contribute to both healthy and diseased states. Examples of free radicals and non-radical oxidants are compiled in Table 1. Several signalling molecules and pathways can be inhibited/activated when oxidative stress disrupts intracellular reduction-oxidation (redox) levels. Those pathways include the nuclear factor erythroid 2-related factor 2/Keap1(Nrf2/Keap1) [21,22,23,24,25,26,27], mitogen-activated protein kinases (MAPKs) [14,28,29,30,31,32], the nuclear factor kappa B (NF-κB) [14,17,33,34,35,36], protein kinase C (PKC) [28,37,38,39,40,41], and phosphoinositide-3-kinase-(PI3K-)Akt [42]. They regulate pro-oxidant genes and antioxidant gene expression, mediating cells’ oxidative injury and antioxidant defence system. Oxidative and nitrosative stress also plays a crucial role in the immune system of pigs, impacting their health and disease resistance. Free radicals can impair immune responses and induce inflammation, affecting overall health, fertility, immunity, and productivity in pigs, leading to economic losses for farmers [43,44]. Therefore, understanding and managing oxidative stress is essential for maintaining optimal immune function and health in pigs.

To maintain the optimum health and well-being of animals, antioxidants serve as the first line of defence against free radical damage [46,47]. Antioxidants are molecules that neutralise ROS and protect cells from oxidative damage [18]. These include enzymes such as superoxide dismutase, catalase, glutathione peroxidase, and non-enzymatic antioxidants like vitamins C and E, glutathione, and flavonoids [48]. Dietary interventions with antioxidants, such as vitamins, can help mitigate oxidative stress and improve animal performance and the quality of meat and milk products [46,49,50].

Vitamins are organic substances required in small amounts for the maintenance and growth of living organisms [51]. They are groups of highly complex compounds present in foodstuffs in traces and essential for normal metabolism. The absence of these nutrients causes disorders. In contrast, resupplying these nutrients can cure the deficiency symptoms [52]. Animal and human body functions depend on vitamins for average growth, nerve function, bone development, and the repair of damaged tissue. Vitamins are vital components of enzyme systems that assist chemical reactions inside the body. They are necessary for the integrity of cell membranes, nerve and muscle function, bone formation, and overall good health [53]. Different species have varying dietary needs for vitamins, some being metabolic essentials while others can be synthesised from other food or metabolic constituents. Swine and other monogastric animals rely more on dietary sources of vitamins than ruminants. Several factors influence vitamin requirements and utilisation, including physiological makeup and production function; confinement rearing without pasture; stress, disease, and adverse environmental conditions; vitamin antagonists; use of antimicrobial drugs; and body vitamin reserve [54,55]. This specific group of substances are differentiated from others by their organic nature, and their classification depends on chemical nature and function [53]. Thus, the classification of vitamins is divided into two main categories: water-soluble and fat-soluble vitamins. B complex vitamins and C are water soluble, whereas vitamins A, D, E, and K are fat soluble. Fat-soluble vitamins are associated with fats and are absorbed by dietary fats. Fat-soluble vitamins’ absorption follows the same mechanics as the absorption of fats. Water-soluble vitamins are not associated with fats and are unaffected by fat absorption alterations [55].

Adding vitamins to animals’ diets can contribute to reducing stress, which may be a solution to improve their welfare [56]. Organisations such as FEDNA, IRTA, and NANP develop recommendations for vitamin supplementation in pigs [57,58,59]. These recommendations are crucial to ensuring optimal animal health, growth, and performance. They take into account many factors, including age, weight, life stage, activity level, and nutritional needs of a particular group of pigs. Those recommendations allow farmers to finetune their pig’s diet to maintain optimal levels of vitamins and other compounds in animals and achieve good production results. However, numerous reports indicate that additional supplementation, above recommended levels, may be helpful in reducing the negative effect of stress induced by various stress factors often present in industrial breeding. Therefore, this paper reviews available studies evaluating the impact of additional supplementations of vitamins on mitigating the effects of various stress factors in pig breeding. 

## 2. Vitamins

### 2.1. Vitamin A

Vitamin A, an essential fat-soluble nutrient crucial for various physiological functions [60], is found in animal and plant sources [61]. It exists in forms such as retinol and provitamin A carotenoids, including *β*-carotene [62]. Three chemical compounds—retinol, retinal, and retinoic acid—play distinct roles in the body, of which the most important biologically active metabolite is retinoic acid [60,63]. In monogastric feed, vitamin A primarily exists as a retinyl ester. Dietary vitamin A esters undergo conversion to retinol, which is then transported to tissues for further metabolism into active forms like retinoic acid [64]. Vitamin A’s role extends beyond vision, supporting cell growth [65] and cell differentiation and exhibiting anti-inflammatory properties by regulating both innate and adaptive immune responses [66,67]. Its contribution to maintaining epithelial barriers prevents inflammation and infections. Vitamin A deficiency impairs cellular immunity and antibody production and increases infection susceptibility [68,69]. In non-ruminants., hypovitaminosis A affects gut-associated lymphoid tissues, leading to gut-related health issues and compromised growth [64]. However, vitamin A supplementation enhances immune responses in non-ruminants, boosting immune cell proliferation, antibody production, and cytokine secretion, thereby improving immune function and disease resistance [70]. Thus, vitamin A supplementation holds promise as an immuno-micronutrient for enhancing health and disease prevention in swine. Nonetheless, there is an ongoing debate and a need for further research regarding the varied impacts of supplemental vitamin A on various facets of the immune system in pigs [69].

Vitamin A supplementation in pigs has been shown to mitigate the impact of various stressors. Studies have indicated that vitamin A supplementation can positively influence the immune response to infections [66,67]. Maternal immunisation with virulent rotavirus (RV) and vitamin A supplementation enhances sow immune responses and passive piglet protection against severe diarrhoea. Vitamin A deficiency exacerbates RV-induced diarrhoea and viral shedding in piglets, while supplementation improves gut health and reduces viral shedding and diarrhoea severity [71]. Another common infection in swine is Transmissible Gastroenteritis Virus (TGEV) infection. TGEV infection in piglets leads to severe gastrointestinal symptoms and high mortality rates due to enterocyte damage and intestinal barrier impairment. Pu et al. (2022) investigated the effects of all-trans retinoic acid (ATRA), a vitamin A derivative, on TGEV-induced inflammation in pig cells. ATRA attenuates the inflammatory response by inhibiting pro-inflammatory cytokine release, maintaining epithelial barrier integrity, and downregulating critical molecules involved in the immune response pathway [72].

Also, the Langel et al. (2019) group investigated the effect of oral vitamin A supplementation on the immune response against the Porcine Epidemic Diarrhea Virus (PEDV) in pregnant gilts and their nursing piglets. The results showed that vitamin A-supplemented (30,000 IU/day of retinyl acetate) gilts had decreased mean PEDV RNA shedding titters and diarrhoea scores compared to non-supplemented gilts. Moreover, the survival rate of nursing piglets from vitamin A-supplemented gilts was higher than that of piglets from non-supplemented gilts when challenged with PEDV [73].

Another stress factor is an infection with the parasite Ascaris suum, a parasitic roundworm in pigs, which severely threatens swine health. It results in reduced weight gain, inefficient feed conversion, and liver condemnation at slaughter, causing substantial economic losses in the pork industry. Dawson et al. (2009) investigate immune responses in infected pigs and the effects of all-trans-retinoic acid (ATRA) supplementation. ATRA appears to modulate immune and inflammatory responses, potentially enhancing parasite management with vitamin A supplementation [74].

Supplementing pigs with vitamin A during heat stress conditions has been shown to have beneficial effects on their performance and health. Vitamin A supplementation can improve mineral digestibility, correct electrolyte imbalances, and enhance overall resilience to heat stress [75]. In addition to the challenges posed by diseases in swine, the impact of heat-induced stress on pigs further underscores the multifaceted concerns within the swine industry. The paper by Usenko et al. (2020) investigates the effects of vitamins on the prooxidant–antioxidant balance in boars exposed to heat stress conditions. The boar diet was enriched with a vitamin supplement containing vitamins A, C, and E. Rearing boars in elevated temperatures induces heightened peroxidation processes and depletes their blood antioxidant defence system. Supplementing the diet with antioxidant vitamins above standard levels at 10% or 20% led to a notable increase in vitamin A and vitamin E and reduced glutathione. Moreover, introducing a vitamin supplement into the boars’ feed induced significant changes in the state of polycyclic aromatic hydrocarbons (PAH) in their tissue and decelerated peroxidation processes, resulting in a decrease in the concentration of diene conjugates and TBA-active complexes in the blood [76].

As a fat-soluble vitamin, prolonged intake of vitamin A can potentially result in toxic effects [77]. The manifestation of symptoms, however, depends on factors such as species, age, and physiological state. Hypervitaminosis may present with skeletal abnormalities, vomiting, distorted vision, hypercalcemia, and thickened skin [63,77]. Vitamin A toxicity in pigs manifests through various symptoms, including roughened hair coat, scaly skin, hyperirritability, lesions in bones, bleeding from skin cracks around hooves, blood in urine and faeces, loss of leg control, and periodic tremors [78].

In essence, vitamin A supplementation emerges as a valuable strategy for promoting swine health, providing a holistic approach to address infectious agents, parasitic threats, and environmental stressors within the swine industry.

### 2.2. Vitamin B

Vitamin B, a complex of water-soluble compounds, is a group of essential nutrients crucial for various physiological functions in the human body. Comprising eight distinct B vitamins, including B_1_ (thiamine), B_2_ (riboflavin), B_3_ (niacin), B_5_ (pantothenic acid), B_6_ (pyridoxine), B_7_ (biotin), B_9_ (folate), and B_12_ (cobalamin), these micronutrients collectively contribute to energy metabolism, cellular growth, DNA synthesis, and neurological health [79]. B vitamins are essential coenzymes for proper mitochondrial and cellular function, including the metabolism of amino acids, purines, and fatty acids, as well as oxidation-reduction reactions [80]. Each B vitamin plays a unique role in supporting different aspects of bodily function, emphasising the significance of a well-balanced B-complex intake. Thiamine, for example, is a crucial coenzyme for energy metabolism and nerve function, folate is essential for DNA synthesis and cell division, and vitamin B_12_ is vital for red blood cell formation and neurological health [79,81,82]. In addition to their fundamental roles, some B vitamins exhibit anti-inflammatory properties, contributing to the body’s immune response and overall well-being. For instance, vitamin B_6_ plays a role in modulating inflammation by influencing cytokine production, while niacin has been associated with anti-inflammatory effects by affecting immune cell function [82,83].

Weaning and gestation are pivotal stages in the reproductive cycle of sows, crucially influencing their overall well-being and productivity. However, these periods often serve as stress inducers for the sows due to various factors [9,10]. Weaning stress is closely linked to immune and intestinal barrier issues, induces oxidative stress through damage to the oxidation–antioxidant system, and contributes to gut microbiota dysbiosis, limiting growth, inducing diarrhoea, and heightening the risk of gastrointestinal diseases in piglets [84,85,86]. Various studies have explored the effects of different compounds from the group of B vitamins on pigs’ growth and reproductive performance with mixed results [78]. For instance, the addition of folate at doses of 9 and 18 mg/kg in post-weaning piglets has been shown to increase organ weight, digesta acidity, the concentration of short-chain fatty acids (SCFAs), and the composition of intestinal microbiota [87]. Similarly, supplementing folic acid in the diet of lactating sows with amounts ranging from 12.5 to 100 mg/kg has been linked to improvements in piglet average daily gain (ADG), weaning weight, and litter weight. These benefits are believed to be due to increased milk production and better milk quality resulting from folic acid supplementation. Additionally, folic acid in sow’s milk directly promotes the growth and development of piglets [88]. Considering reproductive aspects, Almubarak’s study investigated the impact of Niacin treatment (300, 600, and 900 μM) during in vitro maturation (IVM) on porcine oocyte nuclear maturation and subsequent in vitro embryo development [89]. Niacin supplementation during IVM reduced ROS levels in porcine oocytes, suggesting a potential role in alleviating oxidative stress. Additionally, niacin treatment during IVM contributed to increased glutathione (GSH) levels in oocytes, a crucial antioxidant for cell protection against oxidative stress. In cumulus cells, niacin treatment resulted in a significant upregulation of the antiapoptotic gene *BCL2* and a downregulation of the proapoptotic gene *BAX*. The expression of BAX was significantly lower in oocytes, while *BCL2* showed a slight increase in niacin-treated oocytes, though the difference was not statistically significant. In conclusion, the study suggests that supplementing porcine IVM medium with Niacin enhances the developmental competence of early embryos by mitigating oxidative stress and influencing the regulation of apoptosis [89].

Appropriate protein levels in the diet are crucial for healthy growth. The effect of vitamin B_6_ supplementation was tested on weaned piglets fed with a low-protein diet [90]. Such a diet may affect intestinal morphology and function by regulating the aminoacid metabolism. It was shown that additional supplementation with 4 mg/kg of vitamin B_6_ down-regulated the mRNA expression of inflammatory cytokines such as interleukin-10 (*IL-10)*, transforming growth factor beta (*TGF-β)*, and cyclooxygenase-2 (*COX-2)* in the jejunum and up-regulated the expression of *IL-1β*, tumour necrosis factor alfa (*TNF-α)*, *COX-2*, *IL-10*, and *TGF-β* in ileum, which implies that the vitamin could play a role in immune signal transduction, regulate the differentiation of immune cells, and influence the production of cytokines [90]. Also, a high-protein diet can have adverse effects, often leading to undigested gut protein fermentation. This fermentation process can produce potentially harmful metabolites, which may compromise epithelial integrity, which facilitates the invasion of bacteria, toxins, and pathogenic microorganisms, leading to intestinal inflammation, reduced nutritional absorption, and diarrhoea [91]. The addition of 7 mg/kg of vitamin B6 in feed tends to decrease diarrhoea rate, increase villus height and width, increase the expression of *TNF-α*, *COX-2*, *IL-10*, and *TGF-β* in ileal, and change the mRNA expression of a few AAs transporters, suggesting that additional vitamin B_6_ can affect intestinal morphology and metabolism of proteins in weaned piglets fed with a high-protein diet [92]. One of the entomopathogens that affects pigs, causing severe diarrhoea symptoms and high mortality rates, especially among newborn piglets, is Porcine Deltacoronovirus (PDCoV) [93]. Adding 40 mg of niacin has been shown to alleviate the symptoms of PDCoV infection, namely diarrhoea, intestinal barrier damage, intestinal immune response, and microflora dysfunction. Niacin can potentially mitigate the inflammatory response by suppressing the activation of the TLR2/TLR4-NF-κB signalling pathway and modulating histone acetylation via GPR109A in the intestinal tract [94]. 

Various diseases affect the skin of pigs and can be infectious (bacterial, viral, mycotic, and parasitic) or non-infectious (environmental, nutritional, hereditary, and neoplastic) [95]. Those skin diseases in pigs can negatively impact production due to treatment costs, growth retardation, or even death of the animal [96]. Porcine skin is a valuable animal model for exploring treatments for human conditions. The study by Zhao et al. (2022), using the porcine model, aimed to investigate the therapeutic potential of vitamin B_12_ ointment in addressing radiodermatitis, a common side effect of cancer radiotherapy, lacking consensus on effective treatments [97]. It was shown that 12 weeks of vitamin B_12_ treatment significantly alleviated radiodermatitis and notably reduced NF-κB, COX-2, IL-6, and TGF-β expression levels in the skin samples. It might be concluded that vitamin B12 promotes tissue repair, showing anti-radiation, anti-inflammatory, and anti-fibrosis effects [97].

Various B vitamins have been studied for their effects on alleviating stress effects in pigs, pig growth, and reproduction, showing mixed outcomes. For example, folate and folic acid supplementation in pig diets has improved piglet growth and lactating sow performance. Niacin treatment during in vitro maturation of porcine oocytes reduced oxidative stress and influenced apoptosis regulation, enhancing embryo development. Vitamin B_6_ supplementation in weaned piglets down-regulated inflammatory cytokines in the jejunum but up-regulated in the ileum, suggesting a complex role in immune modulation. Additionally, niacin supplementation alleviated symptoms of PDCoV infection, while vitamin B_12_ ointment demonstrated anti-inflammatory effects in pigs with radiodermatitis. Vitamin B toxicity is rare, and no such reports have been reported in swine; therefore, this group of compounds emerges as a potential therapeutic application in various conditions in pigs [78].

### 2.3. Vitamin C

Vitamin C (L-ascorbic acid) is a water-soluble vitamin. It acts as a potent antioxidant [98], protecting the body from damage caused by free radicals [99]. It plays a key role in collagen synthesis and is an essential component of collagen production [100]. The tissues with high metabolic activity contain large amounts of ascorbic acid. It is mainly concentrated in the adrenal glands and nuclei. Adrenal glands are less able to produce ascorbic acid when exposed to stress. When stressors are removed, they quickly return to baseline levels [101,102]. Pigs usually produce enough Vitamin C for their needs. But when pigs are under stress, they may need extra vitamin C [103].

Vitamin C has been shown to play a crucial role in mitigating stress in pigs. Studies have demonstrated that vitamin C supplementation can help regulate ROS synthesis [104] and alleviate oxidative stress induced by various stressors like heat stress [105,106] and mycotoxin-induced stress [107]. Vitamin C has been observed to protect piglet livers against oxidative stress induced by mycotoxins like zearalenone (ZEN). Zearalenone, a mycotoxin found in contaminated feed, has been shown to disrupt early pregnancy in swine by reducing progesterone levels [108]. Pig zearalenone exposure can also lead to metabolic disruptions, affecting lipid and glucose metabolism and altering circulating adipokine concentrations like adiponectin and resistin [109]. Furthermore, zearalenone has been linked to immune response alterations in young pigs, impacting pro-inflammatory and anti-inflammatory cytokines and molecules in the liver’s inflammatory processes [110]. In the case of mitigating effects of vitamin C on the toxicity of zearalenone in diets fed to weaning piglets, adding 150 mg/kg of vitamin C to the ZEN-containing diet prevented vulva deformities, preserved immune response capacity, maintained serum biochemical indicators within normal ranges, and prevented disruptions in hormone levels compared to piglets fed only the ZEN-containing diet. These findings suggest that supplementing diets containing ZEN with vitamin C can alleviate the toxic effects on piglets, including deformities, immune response, serum biochemical indicators, and hormone levels [111]. Other studies demonstrate that vitamin C protects weaning piglet livers from oxidative stress caused by ZEN by regulating nuclear receptors PXR (pregnane x receptors) and CAR (constitutive androstane receptor). This process increases enzyme gene levels, reducing ZEN residues and improving oxidative stress markers. Additionally, vitamin C reduces the liver coefficient and the accumulation of ZEN residues in the liver while enhancing the levels of oxidative stress markers such as MDA (methane dicarboxylic aldehyde), SOD (superoxide dismutase), T-AOC (total antioxidant capacity), and GSHPxThus (glutathione peroxidase). Vitamin C acts as an antioxidant, potentially reducing ZEN contamination in food and feed products [107].

Heat stress in swine can lead to various physiological changes, including alterations in antimicrobial resistance [112], mineral digestibility, and electrolyte balance [105], as well as impacts on lipid profiles and oxidative stress markers [113]. Specifically, in the case of heat stress, vitamin C supplementation, along with other vitamins and micro-minerals, has been shown to improve growth and meat quality parameters in pigs exposed to heat stress conditions. Adenkola et al. (2004) found that vitamin C may help regulate pig body temperature during the harmattan season, potentially mitigating harmattan stress effects [114]. Other studies showed decreased vitamin C levels in pigs with classical swine fever, highlighting the relevance of antioxidant supplementation in managing oxidative stress in affected pigs [43]. Next, studies investigated vitamin C’s antioxidant properties during kidney reperfusion after warm ischemia in a porcine in vitro model. Vitamin C significantly reduced oxidative stress, increased antioxidant capacity, and improved red blood cell protection and haemoglobin concentrations compared to the control [115].

Regarding the impact of adding vitamin C to pig diets on antioxidant levels and meat quality, several studies have observed improvements in both swine growth and the quality of meat [116,117,118] with vitamin C supplementation. Mourot et al. (1990) suggested that alterations in glucose and glycogen metabolism, potentially influenced by vitamin C, could improve pork quality [117]. Oxalic acid, a byproduct of ascorbic acid, may inhibit glycolysis, reducing postmortem lactic acid production and the associated pH drop [119]. Moreover, vitamin C has been found to mitigate preslaughter stress responses by inhibiting glucocorticoid synthesis, further affecting glucose and glycogen availability for lactic acid production [118]. Lahučký et al. (2004) studied the impact of dietary vitamin C on pig antioxidative status and meat quality. While supplementation increased L-ascorbic acid levels in fresh and chill-stored meat, no significant differences were found in cooked and frozen meats. However, using the Thiobarbituric Acid Reactive Substances (TBARS) method, no additional effect of vitamin C on the oxidative stability of these meat types was observed [120].

Adding vitamin C to the nutrition of farm animals is primarily motivated by the desire to mitigate the effects of exposure to stressors such as weaning. Supplementation with vitamin C and vitamin E post-weaning improves oxidative status and immune response in piglets, reducing the incidence of diseases [121]. Studies show no improvement in growth rate, feed intake, or feed conversion was observed with vitamin C supplementation [122]. In contrast, Aznar et al. (2024) show that supplementation with vitamin C positively affected piglets’ birth weight [123]. Other studies showed that supplementing sows with vitamin C increased throughput by enhancing piglet birth weight, weaning weight, and offspring growth performance [124]. Supplementing sows’ feed with higher levels of vitamin C during the late stage of gestation and lactation resulted in elevated levels of these vitamins in the serum of sows, their milk, and the serum of piglets. However, there was no apparent beneficial effect of increased vitamin C supplementation on the rearing outcomes of piglets. Nevertheless, a positive subsequent impact of the vitamins provided to sows was noted: a combination of vitamin E and C significantly lowered the body temperature of sows following farrowing and notably decreased the number of sows culled after rearing [125]. A study by Lechowski et al. (2016) demonstrates that vitamin C supplementation in gilts and sows improves reproductive performance. This included an increase in the number of corpora lutea and the synthesis of 17b-estradiol. Additionally, it exhibited anti-stress effects and enhanced the immune system, resulting in higher levels of immunoglobulins in colostrum and milk. As shown in previously described research, supplementing vitamin C positively affects the body weight of piglets [126].

Earlier investigations into the effects of vitamin C supplementation on litter size, such as those conducted by Yen and Pond (1983) [127] and Chavez (1983) [128], involved administering vitamin C to large groups of sows for one week before farrowing. However, regardless of the dosage (1 g/day, 2 g/day, or 10 g/day), these studies found no significant impact on litter size, stillborn numbers, or piglet weight at birth.

In conclusion, Vitamin C supplementation in pigs has been shown to mitigate the effects of stress. It acts as a potent antioxidant, regulating reactive oxygen species synthesis and improving immune response during stressful conditions such as heat stress and mycotoxin exposure. Additionally, vitamin C supplementation reduces post-farrowing stress in sows and decreases culling rates. However, its impact on litter size remains debated, with conflicting findings in studies. Overall, vitamin C plays a crucial role in managing stress and enhancing the well-being of pigs in various production environments. In principle, it is impossible to overdose on vitamin C taken in dietary form. At the same time, it cannot be said that it is impossible. Large doses of ascorbic acid are fortunately not toxic. The compound dissolves in water, and the excess is excreted from the body with urine within a few hours.

### 2.4. Vitamin D

Vitamin D, a fat-soluble secosteroid, plays a crucial role in various physiological functions [129]. The two primary forms of vitamin D are vitamin D_2_ (ergocalciferol), found in plant-based sources, and vitamin D_3_ (cholecalciferol), found in animal-based sources and synthesised endogenously in the skin upon exposure to ultraviolet B (UVB) radiation [129]. Primary forms of vitamin D undergo conversion into calcidiol (25-hydroxyvitamin D) in the liver and then further metabolic changes into calcitriol (1,25-dihydroxyvitamin D) in the kidneys, which is the active form of vitamin D [130]. Primarily recognised for its classical functions in calcium and phosphorus homeostasis, vitamin D is intricately involved in bone mineralisation and skeletal health [131]. Beyond its traditional role in mineral metabolism, vitamin D influences a range of physiological processes, including immune modulation, inflammation, oxidative stress, mitochondrial respiration, cell proliferation, and processes connected with cardiovascular and reproductive health [129,132,133]. Vitamin D maintains mitochondrial balance, protects against oxidative stress-induced protein, lipid, and DNA damage, and regulates various cellular processes, including autophagy, inflammation, epigenetic modifications, DNA integrity, and calcium and ROS signalling [129]. The presence of vitamin D receptors (VDRs) in various tissues underscores its broad-reaching impact, with research continually unveiling new aspects of its significance [134]. Vitamin D plays a crucial role in pig breeding due to its multifaceted effects on reproductive performance, immune function, bone health, and overall well-being and stress mitigation in both sows and piglets [135].

Weaning induces oxidative stress in piglets, leading to stunted growth and intestinal issues like inflammation and diarrhoea [84,85,86]. Both in vivo and in vitro data support the positive role of vitamin D3 in maintaining normal male reproduction, female fertility, and embryo implantation [136,137]. In pigs, several studies focus on the critical stages of weaning and gestation, acknowledging their impact on sow well-being and productivity. Adding 50 μg/kg 25(OH)D_3_ to sow and progeny diets alongside fortified basal diets can be a supplementation strategy to enhance growth and health in pigs. This supplementation significantly improved pre- and post-weaning growth rates, birth weight, litter size, and feed efficiency [138,139]. Notably, it positively affected muscle gene expression by downregulating myostatin (MSTN) (a negative regulator of muscle mass) and upregulating myoblast determination protein 1 (MyoD) and Myogenic factor 5 (MYF5) (pro-myogenic markers) [139]. Pigs on 25(OH)D_3_ diets exhibited higher serum 25(OH)D_3_ concentrations, improved water holding capacity in meat, reduced drip loss, and lower IL-6 levels, indicating potential health benefits [139]. The plasma 25(OH)D_3_ levels in newborn piglets are intricately linked to maternal levels, suggesting that high concentrations of vitamin D supplementation in sow diets could effectively increase the vitamin D status of the offspring [137,140]. Maternal supplementation of 25(OH)D_3_ improves milk quality and enhances piglet bone strength, density, and ash content [141]. Moreover, additional supplementation increased leukocyte numbers, improved leukocyte survival capacity, and enhanced phagocytic antimicrobial activity, which may have implications for managing weanling pigs susceptible to diseases like the post-weaning respiratory disease complex [142]. Under intensive breeding conditions, piglet bone development relies heavily on calcium (Ca) and phosphorus (P) reserves, influenced directly by dietary levels. Inadequate Ca and P levels or improper ratios impede bone formation. Prolonged exposure to dim light conditions can disrupt vitamin D_3_ production, impairing Ca and P absorption and metabolism, further hindering bone development. The study by Zhang et al. (2022) examined the impact of 25(OH)D_3_ in weaned piglets fed with a low Ca-P diet [143]. It was shown that adding 50 μg/kg 25(OH)D_3_ effectively alleviated average daily gain reduction and decreased immunoglobulins concentrations (IgG and IgA) caused by dietary Ca and P deficiency. The addition of vitamin D also changed faecal microbiota composition, such as a reduction in Streptococcaceae abundance and an increase in Lachnospiraceae abundance and improved bone biochemical parameters, which may subsequently enhance the growth of piglets and improve antioxidant capacity. However, it should be noted that those effects were similar to those of the group fed a normal diet [143]. Also, supranutritional doses of vitamin D supplementation (4999 IU/kg of feed) were tested to assess its effect on the immune development of suckling piglets and their robustness against the *E. coli* challenge [144]. The study suggests that pre-weaning 25(OH)D_3_ provision increases availability post-weaning, potentially beneficial during immune system challenges. The experiment demonstrates vitamin D utilisation during immune activation, observed through lower 25(OH)D_3_ concentrations in *E. coli*-inoculated pigs. Despite reduced immunological responses, 25(OH)D_3_-supplemented pigs showed improved resistance to *E. coli* infection, possibly due to enhanced vitamin D bioavailability for immune responses [144]. Typically, vitamin D supplementation involves either vitamin D_3_ or 25(OH)D_3_, with the majority of research conducted on these forms of vitamin D. However, there are also studies investigating the effects of supplementation with the active form of vitamin D—1,25(OH)_2_D_3_. The addition of 260–300 mg/sow/day of 1,25(OH)_2_D_3_ from plant sources increased the litter size and litter weight gain and reduced farrowing duration, weight loss of sows, and the rectal body temperature after farrowing; however, it increased the number of mummies and the number of short ruptured umbilical cords [145,146]. 

Viral infections are among the most common causes of mortality and subsequent economic loss, especially at weaning [137]. The studies show that larger amounts of active vitamin D might be beneficial against viral infections in pigs [147]. One study found that 1,25-dihydroxyvitamin D_3_ (1,25(OH)_2_D_3_) supplementation inhibited the inflammatory response induced by PEDV infection in piglets [148]. PEDV pathogenesis is related to intestinal inflammation, followed by increased cytokine expression and secretion, leading to watery diarrhoea and vomiting. The effects of vitamin D were tested on IPEC-J2 cells. The findings revealed that PEDV infection increased IL-19 and C-C motif chemokine ligand 20 (CCL20) mRNA expression, while 1,25(OH)_2_D_3_ (20nM) pretreatment inhibited these expressions. The study further investigated the underlying mechanisms and demonstrated that 1,25(OH)_2_D_3_ suppressed, through VDR, the NF-κB and JAK/STAT signalling pathways, crucial for inflammatory responses. Interestingly, despite inhibiting the JAK/STAT pathway, 1,25(OH)_2_D_3_ did not compromise PEDV replication. Additionally, the study suggested that 1,25(OH)_2_D_3_ could enhance the antiviral effects of interferon alfa (IFN-α) by increasing p-STAT1 [148]. Another frequent pathogen in pigs is the porcine rotavirus (PRV). The PRV challenge causes a reduction in villus height, a reduction in faecal consistency, a decrease in growth performance, an increase in cytokine production, and a decrease in 1,25(OH)_2_D_3_ serum concentration [149]. It was shown that vitamin D supplementation with 5000 IU attenuated the adverse effects of the PRV challenge, including decreased body weight gain, feed intake, and villus height. Vitamin D supplementation activated the retinoic acid-inducible gene I (RIG-I) signalling pathway in the intestine, as evidenced by the up-regulation of RIG-I, interferon beta (IFN- *β*), IFN- *β* promoter stimulator 1, and interferon-stimulated gene 15 (ISG 15) mRNA expression [149]. The RIG-1 pathway is a crucial component of the innate immune system, activated in response to viral infections, which then activates signal transduction by transcription factors such as interferon regulatory factor (IRF) and NF-κB, leading to the production of interferons and proinflammatory cytokines [147]. 

Feeding constitutes a significant proportion of swine production costs, and its fluctuations directly impact overall profitability. To address this challenge, diversifying raw materials by incorporating by-products and low-grade grain has been considered. However, this approach introduces the risk of mycotoxin contamination, notably deoxynivalenol (DON), a prevalent mycotoxin in swine diets. The Fusarium fungus produces DON and is commonly present in grains such as wheat, barley, oats, millet, and corn. Despite its adverse effects on pig growth performance, particularly at higher doses, pigs exhibit sensitivity to DON, manifesting in altered immune function, oxidative stress, and intestinal damage [150]. A study delved into the impact of DON contamination on piglets and explored the potential role of vitamin D supplementation (200 IU vitamin D_3_/kg/2200 IU vitamin D_3_/kg/2000 IU 25(OH)D_3_/kg) in alleviating these effects [150]. Repetitive exposure to DON disrupted piglets’ vitamin D, calcium, and phosphorus metabolisms. This led to decreased growth performance, increased bone mineralisation, and the downregulation of genes related to calcium and phosphorus absorption in the intestines and kidneys. Additionally, DON contamination decreased blood concentrations of 25(OH)D_3_, 1,25(OH)_2_D_3_, and phosphate in piglets. Interestingly, vitamin D supplementation, whether with vitamin D_3_ or 25(OH)D_3_, did not fully restore vitamin D status or bone mineralisation in the piglets. However, when piglets were subjected to lipopolysaccharide (LPS)-induced inflammatory stimulation, 25(OH)D_3_ supplementation increased 25(OH)D_3_ concentration, and the regulation of 1,25(OH)_2_D_3_, improving the immune response during the DON challenge. Although no effect of vitamin D supplementation alone was observed, the study showed that 25(OH)D_3_ supplementation might induce vitamin D anti-inflammatory response in combined LPS and DON challenge in pigs [150].

Stressful conditions, particularly fasting before slaughter, have been associated with elevated cortisol levels and disturbances in the antioxidant balance, leading to decreased serum α-tocopherol concentrations. Rey et al. (2020) group conducted two experiments to assess the impact of administering supranutritional doses (500,000 IU/L or 700,000 IU/L) of vitamin D_3_ in drinking water during the lairage period before slaughter on physiological stress, oxidative status, and pork quality [151]. The results revealed that vitamin D_3_ supplementation in drinking water significantly mitigated pigs’ physiological stress, evidenced in reduced cortisol levels and increased α-tocopherol concentrations. Furthermore, the supplementation improved oxidative status, as evidenced by lower oxidative stress markers such as MDA and TBARS. The study also suggested positive effects on preserving vitamin E serum levels in fasting conditions when vitamin D_3_ was added to water. Meat analysis from pigs receiving supplementation indicated lower production of free polyunsaturated fatty acids (PUFA), potentially influenced by higher vitamin E concentrations, contributing to enhanced lipid stability. Texture profile analysis indicated enhanced cohesiveness, gumminess, and chewiness in the vitamin D-supplemented group compared to the control, implying favourable effects on texture parameters under fasting conditions. In summary, administering vitamin D_3_ to fasting pigs’ drinking water at 500,000 IU/L for 4 h alleviates physiological stress, enhances α-tocopherol levels in serum and muscle, improves oxidative status and lipid stability, and positively influences drip loss and tenderness [151].

Pigs have emerged as a valuable animal model for various medical conditions, owing to their physiological and anatomical similarities to humans. For instance, Rai et al. (2021) evaluated the effect of different vitamin D statuses on immunomodulation in the neointimal tissue of the coronary artery in microswine [152]. Animals were first fed a high cholesterol diet, and then intimal injury was induced. The decreased expression of IL-33, macrophages, and neointimal area in the vitamin D sufficient (1500 IU/day) and supplemented (3000 IU/day) group supports the conclusion that vitamin D supplementation has a therapeutic effect in reducing inflammation and neointima formation post-vascular intervention [152]. On a similar model of atherosclerotic microswine, the Gunasekar et al. (2018) group investigated the impact of different vitamin D statuses on regulating inflammation in the epicardial adipose tissue (EAT). They found that vitamin D deficiency increased the number of pro-inflammatory M1 macrophages in the EAT, while vitamin D supplementation promoted the presence of anti-inflammatory M2 macrophages. The shift in the M1:M2 ratio is associated with decreased pro-inflammatory cytokines (TNF-α and MCP1) in EAT. The study suggests that vitamin D impacts the inflammatory processes and cytokine expression, emphasising its potential role in cardiovascular health [153]. 

In conclusion, vitamin D supplementation in swine has proven to be a versatile and beneficial strategy across various aspects of health and performance. The findings underscore the potential of vitamin D supplementation in optimising swine nutrition, particularly in the face of stressful challenges such as infections, weaning stress, and adverse environmental conditions. Furthermore, these observations suggest that dietary 25(OH)D_3_ proves more effective than the commonly used vitamin D_3_, particularly in promoting reproductive performance and preventing infections [135,138,139,141,142,143]. It should be noted that despite the potential positive effects of vitamin D in pig farming and its low risk of natural overdose, excessive supplemental doses can be harmful. Prolonged intake of high doses of vitamin D supplements can lead to toxicity, resulting in hypercalcemia, soft tissue mineralisation, reduced feed intake and growth rate, or even death [78,154,155]. 

### 2.5. Vitamin E

Vitamin E has been studied for its physiological properties and anti-oxidative effects for nearly a century [156]. Vitamin E is a fat-soluble compound made up of tocopherols and tocotrienols [157]. This vitamin constitutes a group of eight naturally occurring compounds, encompassing four tocopherols (α, β, γ, and δ-) and four tocotrienols (α, β, γ, and δ-), each possessing distinct biological functions and antioxidative properties. Among these, α-tocopherol stands out as the most potent form, prevalent in various natural sources and frequently employed in vitamin supplementation [158]. Vitamin E is the major antioxidant beneficial for the organism because it reduces oxidative stress and influences cytokine expression [159]. It prevents the body from being damaged by free radicals, highly reactive and destructive compounds formed by the oxidative degradation of polyunsaturated fats [51]. As an essential nutrient, it maintains skin health, immune function, and neurological well-being [159]. 

Vitamin E, an essential nutrient for pigs during weaning, has been shown to affect intestinal morphology and functions by inhibiting jejunal epithelial cell proliferation [160]. Maternal supplementation of vitamin E in the sow diet during gestation and lactation has been found to improve the weight of piglets at weaning and enhance humoral immune function and antioxidant activity in sows and piglets [161]. Supplementing sows’ diets with vitamin E has been found to alter the composition of their colostrum and milk, boosting the oxidative health of their piglets. This may contribute to enhanced gut health and growth in the early weeks of life [121]. Studies indicate that vitamin E supplementation can positively impact intestinal health, immune function, and antioxidant activity during weaning while also improving the composition and stability of sow milk. In a study by Silva-Guillen (2020), vitamin E (α-tocopherol) supplementation increased serum vitamin E levels and improved antioxidant capacity in weaned pigs fed peroxidised lipids. However, it did not significantly enhance growth performance or overall oxidative status [162]. Other studies conducted by Wang et al. (2017) found that high-dose vitamin E supplementation in sow diets during the last week of gestation and lactation positively impacted piglet growth, immune function, and antioxidant activity in both sows and piglets [163]. Amazan et al. (2012) investigated the effects of vitamin E supplementation (d-α-tocopherol) in drinking water and feed for lactating sows and post-weaning piglets. They observed variations in serum α-tocopherol concentrations among piglet groups, influenced by maternal and direct supplementation sources. Additionally, supplementation with natural vitamin E affected ferric reducing antioxidant power (FRAP) and oxidised glutathione (GSSH) levels, indicating alterations in antioxidant capacity. However, immunoglobulin levels in piglet serum remained unaffected by natural vitamin E supplementation [164]. Next, interesting research was conducted by Frakic et al. (2008), who studied α-tocopheryl acetate (a form of vitamin E) in weaned pigs exposed to mycotoxins. They found that vitamin E partially reduced DNA damage in immune cells and enhanced IgG synthesis impaired by toxins, indicating its potential to preserve lymphocyte DNA integrity during mycotoxin exposure [165].

Vitamin E also plays a crucial role in mitigating the adverse effects of heat stress on pigs. Research indicates that supplementing pigs with high levels of vitamin E can help alleviate respiratory alkalosis induced by heat stress [105]. Liu et al. (2018) studied super nutritional vitamin E’s effects on redox status in pigs. They found that while it did not reduce oxidative stress during cyclic heat stress, it did alleviate respiratory alkalosis, suggesting its potential as a summer ration supplement [166]. Additionally, a combination of vitamin E and selenium at elevated levels above recommendations has been shown to improve mineral digestibility, including calcium, selenium, and zinc, despite heat stress exposure [75]. Selenium, in the form of selenocysteine (SeCys) within selenoproteins like selenoprotein P (SelP) and extracellular glutathione peroxidase (GPx3), acts as a vital constituent, shielding cells against oxidative stress by counteracting reactive oxygen species (ROS) [167,168]. It collaborates synergistically with selenium in fortifying the antioxidant defence mechanism [169,170,171]. The dietary requirements for pigs, as outlined by the National Research Committee on Swine Nutrition (2012), vary from 0.3 mg/kg for nursery pigs to 0.15 mg/kg for finishing pigs and breeding sows. The greatest concern in using selenium supplements is that in growing pigs, toxicity can be induced easily by feeding as little as 5 to 10 mg/kg of diet. Signs of toxicity include anorexia, hair loss, liver and kidney damage, edema, hoof loss, and nervous system disorders. However, increasing dietary antioxidants like selenium and vitamin E above standard levels can reduce intestinal leakiness caused by heat stress, highlighting the importance of oxidative stress in compromising intestinal barrier integrity in heat-stressed pigs [75,76,105].

Supplementation with vitamin E has shown beneficial effects on pigs subjected to transport stress. Research has indicated that pigs supplemented with vitamin E exhibited lower cortisol levels during stress periods [172]. Additionally, vitamin E supplementation led to decreased 24 h drip loss values in pigs under transport stress conditions [173]. Furthermore, vitamin E was found to reduce the peak heart rate and improve heart variables related to stress response, suggesting potential sedative and anti-anxiety effects [174]. Overall, the data indicate that vitamin E supplementation can help mitigate the negative impacts of transport stress on pigs by reducing cortisol levels, improving heart rate variables, and enhancing meat quality parameters. The following study connecting the supplementation of vitamin E with a combination of transition metals was conducted by Lauridsen et al. (1999), who analysed dietary vitamin E and copper levels. Researchers found variations in planned versus actual concentrations, especially in fat-rich feeds. They observed increased α-tocopherol with rapeseed oil inclusion and improved growth with copper supplementation initially. Overall, vitamin E enhanced antioxidative status, while copper supplementation, especially at 175 ppm, temporarily boosted growth and feed intake [175].

The generation of cytokines and the resultant metabolic alterations play a crucial role in the body’s adaptive reaction to infections, which is one of the main stress factors. However, it is evident that excessive production of these substances could lead to harmful consequences. Therefore, altering the cytokine production from stimulated macrophages could present a beneficial approach in scenarios characterised by an overabundance of cytokine production [176]. When macrophages encounter endotoxins, they generate reactive oxygen intermediates, such as superoxide. In addition to their recognised harmful effects outside the cell, recent understanding indicates that reactive oxygen species also participate in internal signalling pathways, potentially triggering the activation of various nuclear transcription factors [177]. A study conducted by Webel et al. (1998) showed the impact of a high-dose administration of d-alpha-tocopherol (vitamin E) on pigs subjected to LPS challenge, a constituent of bacterial cell membranes. Pigs pre-treated with vitamin E prior to the LPS challenge exhibited reduced concentrations of IL-6 and cortisol, both recognised as pro-inflammatory cytokines, in comparison to pigs not administered with vitamin E [176]. Other research conducted by Upadhaya et al. (2015) investigated the impact of vitamin E supplementation on proinflammatory cytokine and prostaglandin E2 (PGE2) production in immune system-stimulated pigs. While vitamin E alone did not affect cytokine and PGE2 levels, omega-3 fatty acids independently reduced their production, suggesting their significant role in immune modulation [178].

In conclusion, vitamin E is a crucial antioxidant in pig nutrition, impacting growth, immune function, and stress resilience. While its supplementation in sow diets enhances piglet growth and antioxidant activity, its effects on weaned pigs’ growth performance and oxidative status vary. Additionally, vitamin E shows promise in mitigating heat stress-induced respiratory alkalosis and transport stress effects. Furthermore, interactions with transition metals like copper suggest potential synergistic effects, highlighting its multifaceted role in pig health and nutrition. According to the nutritional requirements of swine, vitamin E is commonly regarded as one of the least toxic vitamins, with no evidence of toxicity reported in swine. High dietary levels, up to 550 mg/kg, have been administered to growing pigs without any adverse effects observed [78].

### 2.6. Vitamin K

Vitamin K, a group of fat-soluble compounds, is crucial in various physiological processes, particularly blood coagulation and bone metabolism. The two primary forms of vitamin K are K_1_ (phylloquinone), obtained from green leafy vegetables, and K_2_ (menaquinone), synthesised by gut bacteria and found in fermented foods and animal products. Vitamin K contributes to the synthesis of clotting factors, ensuring proper blood clot formation and preventing excessive bleeding. Additionally, emerging research indicates its involvement in bone mineralisation, cardiovascular health, and potential anti-inflammatory effects [179,180,181].

Only a few studies are available concerning vitamin K’s influence on pigs, especially in the context of reducing the impact of stress. One study by Wang et al. evaluated the effects of 5 mg/kg vitamin K supplementation on reproductive performance and bone metabolism in sows as a possible way to counteract lameness. Locomotion disability might be affected by various environmental factors, including mechanical stress. One of the results was lower TNF-α on the day of weaning in the supplemented group [182]. TNF-α is a known proinflammatory cytokine, which was also reported to stimulate osteoclast proliferation and suppress osteoblast activity.

## 3. Conclusions

Pigs face a multitude of stressors encompassing environmental, nutritional, chemical, psychological, physiological, and metabolic factors. These stressors include extreme temperatures, transportation, exposure to toxins and pathogens, as well as psychological stress from social integration, fear, or weaning separation. Physiological stressors such as diseases, pregnancy, lactation, and metabolic imbalances like hypocalcemia further contribute to their overall stress burden, particularly in intensive farming systems. The cumulative impact of these stressors disrupts the internal balance of pigs, leading to a state of oxidative stress, which is detrimental to their well-being, causing various health complications and, as a result, economic losses for breeders. Therefore, enhancing stress resilience in pig breeding is crucial for improving animal welfare, productivity, and health. The reviewed literature emphasises the significance of vitamin supplementation, above standard doses, in pig diets to reduce oxidative stress, ultimately enhancing the health and performance of pigs. Research indicates that adding vitamins to swine diets is a flexible and beneficial approach for improving nutrition and addressing specific challenges in swine farming. It is suggested that additional vitamin supplementation should be integrated into pig management practices to promote better health, reproductive performance, and resistance to stressors. A holistic approach to farming, combining proper nutrition with antioxidant supplements, is essential for achieving these goals. Studies highlight the efficacy of vitamins A and D in combating infectious diseases, with vitamins B and D showing significant impacts on enhancing reproductive performance by mitigating stress associated with weaning and/or gestation. Vitamins C and E demonstrate potential in managing environmental stressors like heat stress and toxins. Available studies, along with the used form and dose of vitamin, are available in Supplementary Materials Table S2. While studies show positive effects of supplementing certain vitamins, particularly vitamins E and D, more research is needed to fully understand the effects of different types and forms of vitamins on mitigating stress factors in pigs. It is also extremely important to consider potential toxicity risks associated with vitamin supplementation, especially with fat-soluble vitamins, and to exercise caution when adjusting dosage levels. It should also be noted that studies have shown that different forms of vitamins can have varying impacts on stress factors. For instance, the 25(OH)D_3_ form of vitamin D has been shown to be more effective in improving reproductive health or alleviating the effects of infectious diseases than conventionally used vitamin D_3_. In the case of vitamin E, α-tocopherol had a positive effect on infection-related stress. Moreover, in an analysis of the literature, we can conclude that the combination of different types of vitamins (for example, vitamin E) with micro and macronutrients (for example selenium) has a positive effect on mitigating stress in swine, but future investigations should still be conducted.

In conclusion, implementing additional vitamin supplementation can be a beneficial practice for mitigating the effects of various stressors in modern pig breeding. However, each case should be considered individually and with caution not to induce toxicity when using supranutritional doses, especially in the case of fat-soluble vitamins. Further investigations into additional supplementation in pigs are crucial to better understanding the effects and underlying biological mechanisms of mitigating stress. 

## Figures and Tables

**Table 1:** Examples of free radicals and non-radical oxidants [18,20,28,45].

Free Radicals and Non-Radical Oxidants	Examples
Reactive oxygen species (ROS)	Hydroxyl radical (·OH), Superoxide radical anion (O_2_^·−^), Peroxyl radical (ROO·), Alkoxyl radical (RO·), hydrogen peroxide (H_2_O_2_), perhydroxyl radical (HOO·), Singlet oxygen (^1^O_2_)
Reactive nitrogen species (RNS)	Nitric oxide (NO·), peroxynitrite anion (ONOO^−^), nitrous acid (HNO_2_), nitrogen dioxyde (NO_2_), nitrosyl anion (NO^−^)
Reactive chlorine species (RCS)	Hypochlorous acid (HClO), nitryl chloride (ClNO_2_)
Reactive sulfur species (RSS)	Thiyl radical (RS·), hydrogen sulfide (H_2_S), persulfides (i.e., GSSH, CSSH)

## Data Availability

Not applicable.

## Supplementary Materials

The following supporting information can be downloaded at: https://www.mdpi.com/article/10.3390/ani14081218/s1, Table S1: Graphical abstract of stress sources, biomarkers, signalling pathways and stress effect in swine; Table S2: Summary of the most important studies about the additional supplementation of vitamins in swine. References [71,72,73,74,76,87,88,89,90,92,94,97,111,121,138,139,141,142,143,144,145,146,149,150,151,152,153,161,163,165] are cited in the supplementary materials.
